# Living Weapons: Biological Weapons and International Security

**DOI:** 10.3201/eid1610.100959

**Published:** 2010-10

**Authors:** Theodore J. Cieslak

**Affiliations:** Author affiliation: Centers for Disease Control and Prevention, Atlanta, Georgia, USA

**Keywords:** bioterrorism and preparedness, biodefense, biological weapons, international security, book review

In the summer of 1996, as a (much younger) Army infectious disease physician, I headed off to a new assignment with the US Army Medical Research Institute of Infectious Diseases (USAMRIID) at Fort Detrick, Maryland. It was a heady time to be entering the niche field of biodefense. Five years before the events of 9/11 and the subsequent “Amerithrax” attacks would become an integral part of the world’s consciousness, USAMRIID was (with the exception of a few veterinary laboratories studying anthrax, brucellosis, Q fever, and similar zoonotic diseases) the “only game in town.” The institute had quietly wrestled with issues of medical biodefense since the United States shuttered its old offensive biowarfare program in 1969. As an assignee to its division of operational medicine, I was enamored of my ability to master the sparse literature and science of the field and soon became an “expert.”

Such luxurious self-confidence is no longer warranted (if it ever was) or even possible. The field has, for better or worse, burgeoned in the decade since 9/11. Hundreds of texts and thousands of scientific articles now address every aspect of the daunting problems of biowarfare and bioterrorism defense, and mastery of such material is beyond the grasp of any one scientist, diplomat, or arms-control expert. Fortunately, several good reviews exist to guide those wishing to gain a basic understanding of these complex issues. Was yet another treatise thus necessary? My initial reaction was an emphatic “no,” until I read Gregory Koblentz’s book, Living Weapons.

Koblentz, deputy director of a graduate degree program in biodefense at George Mason University, tackles the myriad issues surrounding biodefense from a policy perspective. In so doing, however, he puts forth a readable, succinct, yet thorough review of the field. After a crisp introduction to the history of biowarfare, he focuses on the seemingly insurmountable obstacles to nonproliferation efforts aimed at state-sponsored weapons programs: treaty compliance verification, program oversight, and intelligence. He then addresses the parallel problems posed by nonstate actors and terrorists and closes with a prescription for reducing the dangers of uncontrolled biology.

A tour de force of bureaucratic hurdles and treaty-compliance issues would hardly seem compelling reading for all but a few die-hard policy wonks. Not so in the case of Koblentz’s text. Even though his penchant for frequent summary makes for some redundancy, he provides an extremely readable, yet comprehensive, compendium of the implications for national security posed by wayward biology. Moreover, he does so without the ideological bias often found in other treatises on this subject. Specifically, Koblentz delivers a detailed review of the United Nations Special Commission inspections in Iraq and the lessons they provide. He similarly deals with the now-discredited 2002 National Intelligence Estimate, which provided much of the impetus for President George Bush’s decision to invade Iraq. Yet, he analyzes these efforts and their implications for diplomats, policymakers, and arms-control experts without descending into partisan politics or anti-US demagoguery.

In summary, Living Weapons is a thoroughly readable book, filled with enough anecdotes to capture the reader’s interest. But, more importantly, it provides a disturbing yet refreshing look at the myriad obstacles confronting those who would play a role—whether political, diplomatic, or scientific—in attempting to rein in the use of biology in war and terror. It is a must read for graduate students and experts alike.

